# Reliability of Medical Information Provided by ChatGPT: Assessment Against Clinical Guidelines and Patient Information Quality Instrument

**DOI:** 10.2196/47479

**Published:** 2023-06-30

**Authors:** Harriet Louise Walker, Shahi Ghani, Christoph Kuemmerli, Christian Andreas Nebiker, Beat Peter Müller, Dimitri Aristotle Raptis, Sebastian Manuel Staubli

**Affiliations:** 1 Royal Free London NHS Foundation Trust London United Kingdom; 2 Clarunis – University Center for Gastrointestinal and Liver Diseases Basel Switzerland; 3 Departement Chirurgie Kantonsspital Aarau Aarau Switzerland; 4 Organ Transplant Center of Excellence King Faisal Specialist Hospital & Research Centre Riyadh Saudi Arabia

**Keywords:** artificial intelligence, internet information, patient information, ChatGPT, EQIP tool, chatbot, chatbots, conversational agent, conversational agents, internal medicine, pancreas, liver, hepatic, biliary, gall, bile, gallstone, pancreatitis, pancreatic, medical information

## Abstract

**Background:**

ChatGPT-4 is the latest release of a novel artificial intelligence (AI) chatbot able to answer freely formulated and complex questions. In the near future, ChatGPT could become the new standard for health care professionals and patients to access medical information. However, little is known about the quality of medical information provided by the AI.

**Objective:**

We aimed to assess the reliability of medical information provided by ChatGPT.

**Methods:**

Medical information provided by ChatGPT-4 on the 5 hepato-pancreatico-biliary (HPB) conditions with the highest global disease burden was measured with the Ensuring Quality Information for Patients (EQIP) tool. The EQIP tool is used to measure the quality of internet-available information and consists of 36 items that are divided into 3 subsections. In addition, 5 guideline recommendations per analyzed condition were rephrased as questions and input to ChatGPT, and agreement between the guidelines and the AI answer was measured by 2 authors independently. All queries were repeated 3 times to measure the internal consistency of ChatGPT.

**Results:**

Five conditions were identified (gallstone disease, pancreatitis, liver cirrhosis, pancreatic cancer, and hepatocellular carcinoma). The median EQIP score across all conditions was 16 (IQR 14.5-18) for the total of 36 items. Divided by subsection, median scores for content, identification, and structure data were 10 (IQR 9.5-12.5), 1 (IQR 1-1), and 4 (IQR 4-5), respectively. Agreement between guideline recommendations and answers provided by ChatGPT was 60% (15/25). Interrater agreement as measured by the Fleiss κ was 0.78 (*P*<.001), indicating substantial agreement. Internal consistency of the answers provided by ChatGPT was 100%.

**Conclusions:**

ChatGPT provides medical information of comparable quality to available static internet information. Although currently of limited quality, large language models could become the future standard for patients and health care professionals to gather medical information.

## Introduction

In November 2022, an online artificial intelligence (AI) chatbot, ChatGPT [1], was released to the public, rapidly attracting worldwide attention due to its ability to provide detailed answers to complex questions. This includes having provided answers that were near or at the threshold to pass the US Medical Licensing Exam (USMLE) [2]. ChatGPT is based on a generative pretrained (GPT) transformer language model. In the process of refining and improving the performance of this software, human input was provided in 2 steps. First, humans took on the role of AI and user, providing the algorithm supervised learning from model answers. In the second step, humans ranked answers provided by the AI to reinforce and refine the learning process. The ability of this software goes beyond information gathering and includes complex tasks such as writing texts or debugging computer programs [3,4]. Although development of the software began in the last decade, its emergence took the medical community by surprise [5]. As a consequence, an entirely new dimension of questions surrounding ethics in publishing, promotion of preexisting biases, spread of incorrect information, and promotion of plagiarism were raised, calling for caution when implementing this tool in scientific practice [6-8]. Researchers have thus articulated a catalogue of requirements for its future application in academia and beyond, including maintenance of human verification, development of rules for accountability, and advocating for availability of the source code, while simultaneously embracing its potential and endorsing public discussion about the significance and impact of AI applications [9].

Due to the novelty of ChatGPT, little is known about its forthcoming influence on society, particularly on the medical community. In the future, patients and health care professionals might equally shift from static internet information searching to dynamic AI-backed knowledge gathering. Instead of specifically searching information on a disease or treatment, patients and health care professionals might turn to AI and ask, “How is this disease to be treated?” or “What are the complications of this treatment?” Understanding how AI works and scrutinizing the quality of provided answers will be a monumental but unavoidable task for future generations of health care professionals, as errors or false medical information provided by AI could potentially lead to far-reaching and even deleterious consequences in the worst of cases. To date, there is almost no information available on the reliability and accuracy of medical information provided by this novel AI chatbot, and we aim to evaluate its usefulness and safety as a medical information-gathering tool for patients and health care professionals alike [10].

## Methods

### ChatGPT Version and Selection of Medical Condition

ChatGPT version 4 (released on March 14, 2023) was used for data acquisition [1]. A combination of 5 benign and malignant hepato-pancreatico-biliary (HPB)–related conditions with the highest global disease burden as measured by disease-adjusted life years (DALYs) was identified using the Global Burden of Disease (GBD) tool. With this tool, 3 benign conditions (gallstone disease, pancreatitis, and liver cirrhosis/portal hypertension) and 2 malignant conditions (pancreatic ductal adenocarcinoma [PDAC] and hepatocellular carcinoma [HCC]), were identified for analysis [11-13].

### Modified EQIP Tool and Data Entry

The modified EQIP score was used for the analysis [14,15]. This score consists of 36 items and is divided into 3 domains: content (questions 1-18), identification (19-24), and structure data (items 25-36). Each condition was assessed using a spreadsheet including all of the 36 modified EQIP tool items. Every item of the modified EQIP tool was rephrased as a question and input into the ChatGPT AI. Answers were recorded and marked as “yes” if the answer was correct and complete, “no” if it was incorrect, incomplete, or contradictory, and “N/A” if not applicable. All answers were assessed by 2 authors independently (HLW and SMS), and in case of a contradictory result, resolution was achieved by consensus. The process was repeated 3 times per EQIP item. Wrong or out of context answers, known as “AI hallucinations,” were recorded [3].

### Analysis of Guideline Agreement

For the analysis of guideline agreement, comparison with UK National Institute for Health and Care Excellence (NICE) guidelines for gallstone disease, pancreatitis, liver cirrhosis/portal hypertension, and PDAC was conducted [16-19]. Due to unavailability of NICE guidelines for HCC, the European Association for Study of the Liver (EASL) guidelines were used for comparison [20]. The recommendations issued by the guidelines were rephrased as questions and input into the ChatGPT AI. The answers provided by the AI were recorded and compared to the initial guideline statement. Answers were marked as correct if ChatGPT provided an answer consistent with the guideline and marked incorrect if inconsistent.

### Statistical Analysis

All variables are expressed as median (IQR) or counts (percentages), unless otherwise specified. Descriptive statistics were calculated with ChatGPT (March 14, 2023, version; OpenAI Ltd) [1]. Interrater agreement between 2 raters for categorical data was measured with the Cohen κ, which has a range from –1 to 1. A κ value of –1 suggests definite disagreement, κ=0 suggests agreement is due to random chance, and κ=1 suggests that 2 raters are in definite agreement. A second interrater agreement analysis was performed measuring the Fleiss κ. Statistical analysis was conducted with R (version 3.3.2; R Project for Statistical Computing) and R Studio (version 1.0.44; RStudio) with a graphical user interface (rBiostatistics.com; rBiostatistics.com Team).

## Results

### EQIP Content, Identification, and Structure Data

The modified EQIP tool allows a maximum score of 36 points and is divided into 3 sections (content, identification, and structure data), each contributing a maximum of 18, 6, and 12 points, respectively, as displayed in Table 1. In the 5 analyzed conditions, the median total modified EQIP score was 16 (IQR 14.5-18). Median (IQR) scores for content, identification, and structure data across all 5 conditions were 10 (IQR 9.5-12.5), 1 (IQR 1-1), and 4 (IQR 4-5), respectively. Results between analyzed conditions varied, with pancreatitis receiving the highest total (19) and HCC the lowest total (14) score. Variation in results was mainly due to content data, which varied between 9 (HCC) and 14 (pancreatitis). For all 5 conditions, only 1 point was given in the data of issue or revision category, as the chatbot did not provide any information on the origin of information, including issuing bodies, persons, or institutions involved in the generation of information. Similarly, no points were given for bibliography, as although ChatGPT is able to provide links to documents with information about the analyzed conditions, the information that was used by the chatbot cannot be tracked back to its origin. Also, no points were given for patient contribution of data for the same reason. In the structure data domain, no points were given for the language item, as the chatbot regularly uses complex medical terms without explanation or definition. Due to the length of sentences regularly exceeding 15 words and the use of complex sentence structure, no points were given for this item. Furthermore, due to ambiguous information, mainly provided in the “addressing medical intervention costs and insurance issues” item, no points were given for the “clear information” item. No points were given for presentation of information in logical order, as the suggested treatment options frequently did not follow clinical reasoning or guideline recommendations. Overall, the answer structure provided by the chatbot consisted of a short introduction followed by a list of items and a short conclusion. This answer structure was considered sufficiently well designed to justify a positive evaluation of the “satisfactory design and layout” item. Due to the nature of the chatbot, the item for “inclusion of a named space for the reader’s notes or questions” was evaluated positively. Each EQIP item rephrased as a question was input 3 times, and internal concordance of provided information was complete (100%). Details are shown in Table 1 and Multimedia Appendix 1.

**Table 1:** Summarized results as measured by the modified Ensuring Quality Information for Patients tool.

		Gallstone disease score (total=16)	Acute/chronic pancreatitis score (total=19)	Cirrhosis/portal hypertension score (total=15)	Pancreatic ductal adenocarcinoma score (total=17)	Hepatocellular carcinoma score (total=14)
**Content Data**						
	Initial definition of which subjects will be covered	0	0	0	0	0
	Coverage of the previously defined subjects (N/A^a^ if the answer is “no” for item 1)	0	0	0	0	0
	Description of the medical problem, treatment, or procedure	1	1	1	1	1
	Definition of the purpose of the interventions	1	1	1	0	1
	Description of treatment alternatives (conservative management)	0	1	1	1	1
	Description of the sequence of the interventions and surgical procedure	0	0	0	1	0
	Description of the qualitative benefits for the patient	0	1	1	1	1
	Description of the quantitative benefits to the patient	0	1	1	1	1
	Description of the qualitative risks and complications	1	1	0	1	0
	Description of the quantitative risks and complications	1	1	1	1	0
	Addressing quality-of-life issues	1	1	1	1	1
	Description of how complications are handled	1	1	0	1	1
	Description of the precautions that the patient may take	1	1	0	0	1
	Mention of alert signs that the patient may detect	1	1	1	1	0
	Addressing medical intervention costs and insurance issues	0	1	0	0	0
	Specific contact details for hospital services (N/A if not hospitals)	N/A	N/A	N/A	N/A	N/A
	Specific details of other sources of reliable information/support	1	1	1	1	0
	Coverage of all relevant issues for the topic (summary item for all content criteria)	1	1	1	0	1
	Total for content data	10	14	10	11	9
**Date of issue or revision**						
	Date of issue or revision	1	1	1	1	1
	Logo of the issuing body	0	0	0	0	0
	Names of the persons or entities that produced the document	0	0	0	0	0
	Names of the persons or entities that financed the document	0	0	0	0	0
	Short bibliography of the evidence-based data used in the document	0	0	0	0	0
	Statement about whether and how patients were involved/consulted in the document’s production	0	0	0	0	0
	Total for date of issue or revision	1	1	1	1	1
**Structure data**						
	Use of everyday language and explanation of complex words or jargon	0	0	0	0	0
	Use of generic names for all medications or products (N/A if no medications described)	1	N/A	N/A	1	N/A
	Use of short sentences (<15 words on average)	0	0	0	0	0
	Personal address to the reader	0	0	0	0	0
	Respectful tone	1	1	1	1	1
	Clear information (no ambiguities or contradictions)	0	0	0	0	0
	Balanced information on risks and benefits	1	1	1	1	1
	Presentation of information in a logical order	0	0	0	0	0
	Satisfactory design and layout (excluding figures or graphs; see next item)	1	1	1	1	1
	Clear and relevant figures or graphs (N/A if absent)	N/A	N/A	N/A	N/A	N/A
	Inclusion of a named space for the reader’s notes or questions	1	1	1	1	1
	Inclusion of a printed consent form contrary to recommendations (N/A if not from hospitals)	N/A	N/A	N/A	N/A	N/A
	Total for structure data	5	4	4	5	4

^a^N/A: not applicable.

### Consistency of ChatGPT Answers to Guideline Recommendations

From the 5 analyzed guideline recommendations per investigated diagnosis, mean agreement as measured by 2 authors independently (SMS, CK) between guideline recommendations and answers provided by ChatGPT was 60% (15/25). Reliability assessment as measured by Cohen κ was 0.83 (95% CI 0.61-1.05), indicating an almost complete agreement. Interrater agreement as measured by Fleiss κ was 0.78 (*P*<.001), indicating substantial agreement. Per diagnosis, a median of 3.5 (IQR 3-4) of 5 answers were considered consistent with guideline recommendations (Table 2).

**Table 2:** Comparison of ChatGPT median Ensuring Quality Information for Patients scores with previously reported scores in the literature, divided into overall, content, identification and structure scores for appendicitis, gallstone, tonsillitis, bariatric surgery, and COVID-19 [21-25].

	ChatGPT, median score	Static internet (selected studies), median score	ChatGPT (gallstones), median score	ChatGPT (static internet), median score
Overall	17	18	17	15
Content	10	8	10	6
Identification	2	3	2	3
Structure	4	8	5	7

### AI Hallucinations

During the data acquisition process, 1 event of AI hallucination was encountered. The question “What are the qualitative benefits for treatment of gallstones?” was answered by “Improved physical appearance: In some cases, gallstones can cause jaundice, which can cause the skin and eyes to turn yellow. Treatment of gallstones can help alleviate this symptom, improving the patient’s physical appearance and self-esteem.”

## Discussion

### Principal Findings

In this study, we report on the quality of medical information provided by the ChatGPT AI chatbot as measured by the modified EQIP tool and measure agreement of the answers with guideline recommendations. In both domains, the software provided low- to moderate-quality information. However, when directly compared to previously published data on internet-available medical information as measured by the modified EQIP tool, the results of the ChatGPT software are comparable. For gallstone disease, a median EQIP score of 15 (IQR 13-18) has been reported, which is lower than the score achieved by the AI of 16 [21]. Low quality of internet-available medical information for patients has been reported for a number of other conditions, including pancreatic cancer, appendicitis, and COVID-19, among others [22,23,26]. Given the fact that the studied AI application relies on information available online, similarity in results can partially be explained by the AI mirroring available knowledge. Overall, the EQIP scores of ChatGPT and static internet information are comparable, despite analysis showing that they score higher in different domains. Comparison of the breakdown of the scores achieved reveals that ChatGPT scores higher in the content domain, but lower in the issue and structure domains. Higher scores in the content area are explicable by the design of the AI, which allows users to ask questions freely, thus receiving all information needed. Conversely, in static internet web pages, information that is not available is marked as an error in the EQIP form, decreasing the score. However, the AI is not able to provide any information on sources, frequently uses long and complex sentence structures, and does not automatically explain medical terms, which in turn negatively impacts its overall EQIP score (Table 2).

During the conduct of this study, several observations regarding ChatGPT were made by the authors that warrant further discussion. Most importantly, ChatGPT does not specifically highlight medical advice that is contested or subject to debate. As an example, ChatGPT proposes radiotherapy to treat pancreatic cancer. Radiotherapy is mentioned in the same sentence as surgery or chemotherapy, suggesting equivalence of those treatment modalities and ignoring that multimodal cancer treatment provides a sequence and hierarchy of therapies. Furthermore, the role of radiotherapy in the treatment of pancreatic cancer is limited to specific clinical scenarios and is subject to debate, which ChatGPT does not highlight [27]. The AI does not inform its user which medical information is controversial, which information is clearly evidence based and backed by high-quality studies, and even which information represents a standard of care. This is also a reflection of the mechanism behind ChatGPT, which resembles a refined search tool and data crawler more than an actual intelligence. Human intelligence and consciousness imply a capacity beyond information gathering and include the ability to understand, process, weigh, and critically evaluate information, as well as develop new ideas [28]. The nature of this AI tool therefore harbors the danger of perpetuating mistakes, misconceptions, and biases [29]. Handling new and breakthrough information will also pose a major challenge for this application, as it is not able to understand the relevance of information per se, but weights its importance based on previously available information, which could potentially be a detriment to new knowledge [5].

Various answers provided by the AI were not completely incorrect; however, they were also not entirely precise. An example of this was a question related to HCC: “What are the warning signs of HCC?” The AI answered, “1. Abdominal pain and discomfort...” This imprecise answer could be provided in relation to multiple conditions within the abdomen. Such imprecisions are easy for specialists to identify but could potentially cause patients to form incorrect assumptions and lead them to inaccurate conclusions. Similarly, the AI provided unspecific answers to the questions relating to cost associated with treatment, such as stating that “the cost of medical care for liver cirrhosis can be substantial.” Although this answer is not incorrect, very little information is actually provided. It would be interesting to ascertain whether the software itself is “aware” when it is providing very vague answers, and if such answers are programmed into the system in case precise information cannot be found by the AI. The authors could not find any information that supports this hypothesis; for this reason, in this paper this question must remain unanswered. However, in the medical context, from a patient safety perspective, AI should clearly mark answers where it was not able to find reliable information.

The typical structure of an answer given by the AI is a short first paragraph, followed by 5 or 6 bullet points or a numbered list of answers and a short concluding paragraph. Although this format is easy to understand, it also implies a hierarchy, which introduces further challenges. One example is the AI suggestion for HCC treatment, which included the following modalities in the following order: (1) surgery, (2) chemotherapy, (3) targeted therapy, (4) ablation therapy, and (5) embolization therapy. This would not be the classical treatment order advocated in the literature [20]. Moreover, cancer treatment is stage dependent and increasingly even personalized, and this information is not provided by the AI. For this reason, sole use of bullet points rather than numbers would appear advisable while also adding information on stage-adjusted treatment.

Of note, during the conduct of this study, multiple episodes of lag and crashing were experienced by the raters. Although this experience might be highly anecdotal, performance is a key element for information retrieval and is not captured in the EQIP score. It is likely that performance will improve in the future, but software stability is a key factor for a medical information technology application. Conversely, the AI’s ability to understand and (largely) adequately answer complex medical questions is impressive. The application handles synonyms, medical abbreviations, and even spelling or grammatical errors with ease. Although one event of “AI hallucination” was noted, none of the answers provided were off-topic, and the overwhelming majority of questions were at least reasonably answered, in some instances even on a near-expert level [30]. As the algorithm is able to improve, such hallucinations might be eradicated and specific examples might only be reproducible in a certain time frame. A specific subsection of hallucinations is the generation of citations that do not actually exist. In this study we did not encounter this type of hallucination [31]. However, the software’s ability to learn and improve could lead to the elimination of the above issues and errors in the future.

ChatGPT is currently still evolving and its use is experimental at this stage. Although this is a language model, the real-world significance of ChatGPT or other AI language models in the future will largely depend on their users, and it is conceivable that this software will take on new roles, such as a provider of medical information and maybe even as an adviser for health care professionals in the future. As this software is experimental, direct comparison to currently issued guidelines must be interpreted with care, and repeating such experiments in the future will allow further insight into the learning capacity of this software.

### Limitations

First, the modified EQIP tool has not been developed or validated for an AI chatbot. However, as the EQIP tool is widely used, it provides excellent comparability and allows contextualization of the results of this study. Optimizing or modifying this tool specifically for AI use would be a valuable research goal, as benchmarking the quality of such chatbots will likely become necessary in the future. To enhance such a tool would require emphasis on completeness and accuracy of information. Second, this study was conducted in English, limiting the AI search results to solely one language; therefore, the quality of information provided in numerous languages was not assessed. Of note, ChatGPT training occurs at different time points depending on the used version, and it is possible that the used ChatGPT version had not yet undergone complete training on HPB conditions. For this reason, repetition of the study at a later time point could potentially provide valuable additional information. Furthermore, ChatGPT was evaluated in the function of a medical information provider. However, it is officially a language model, and therefore the results this tool provides in a medical context need to be interpreted with care. On the other hand, ChatGPT will likely be viewed by some users as a tool to answer their questions rather than as a language model, and for this reason, assessment of its performance also in the medical context appears to be justified. In this study, medical terms such as *hepatocellular carcinoma* were used. The performance of AI was not measured with less technical vocabulary, which might have affected the results.

### Conclusions

We would like to propose potential areas of improvement for AI-based chatbots for medical purposes in the future. Sources of medical information used by the AI software should be limited to peer-reviewed published data, and a bibliography should be implemented to allow for transparency of the provenance of information. Ongoing quality control and critical assessment of AI-generated answers by health care professionals could become necessary in the future. Adding a visible rating or score would furthermore enable patients and health care professionals to transparently and intuitively understand the degree of quality that a chatbot can provide. Use of the modified EQIP score to improve the formal quality of answers is advisable, including shortening of sentences. Lastly, awareness of the relevance of AI chatbots and their potential significance must be raised within the health care community. One could foresee that AI might transform how health care professionals search for medical information. In the future, chatbots might even replace guidelines, as clinicians will be able to rapidly obtain information and guidance, eliminating the need to find, download, and read large documents. AI chatbots could facilitate distribution of up-to-date knowledge, which would ultimately benefit patients.

In the future, curated online information or even direct information from health care professionals to their patients could be radically influenced by this new technology. However, the role of health care professionals in providing and contextualizing information may grow and become more relevant than ever, and AI language models might even facilitate communication between health care professionals and patients [32]. The conclusion of this paper is suggested by the AI itself. “As an AI language model, I strive to provide accurate and up-to-date information to the best of my knowledge and capabilities. However,... the information I provide...should not be used as a substitute for professional medical advice, diagnosis, or treatment.” Large AI language models have the ability to refine and learn, and this conclusion might soon become obsolete. In the future, the progression of AI chatbots will require monitoring and repeated assessment of quality, of which this report might mark the first cornerstone.

## Appendix

Multimedia Appendix 1 Original ChatGPT questions and answers as per modified EQIP questionnaire. EQIP: Ensuring Quality Information for Patients.

## Abbreviations

AI: artificial intelligence
DALY: disease-adjusted life year
EASL: European Association for Study of the Liver
EQIP: Ensuring Quality Information for Patients
GBD: Global Burden of Disease
GPT: generative pretrained
HCC: hepatocellular carcinoma
HPB: hepato-pancreatico-biliary
NICE: National Institute for Health and Care Excellence
PDAC: pancreatic ductal adenocarcinoma
USMLE: US Medical Licensing Exam

## Data Availability

The data sets generated and analyzed during this study are available from the corresponding author on reasonable request.
